# Supplementary material for: ‘College choice’ under the COVID-19 pandemic: Sustainability of engineering campuses for future enrollments
Source: PLoS One. 2023 Sep 27;18(9):e0291876. doi: 10.1371/journal.pone.0291876 (PMC10530044; doi:10.1371/journal.pone.0291876)
Supplement: Attachment — Submitted filename: Response to Reviewers.docx [file pone.0291876.s001.docx]

**RESPONSE TO REVIEWERS**

Date 17.06.2023

Respected Reviewers

First and foremost, thank you for reviewing our research paper. Your inspiring remarks have enlightened us. Further, we are grateful to you for offering helpful suggestions and recommendations that helped make this updated paper more constructive.

Following are corrections/additions made in a manuscript as per your directions/suggestions.

**Reviewer#1:** No specific requirement for revision

**Reviewer#2:** Following matter / text added to improvise the manuscript

**Comment (1)**

“In the theory please describe more about relations between sustainability and COVID pandemic. How the pandemic has impacted various problems connected with sustainability?”

**Response by authors**

As per reviewer’s suggestion following text is added

“Sustainability in education is all about designing practices that can be scaled or right-sized without depleting resources or exclusion of groups (301). Sustainability became more important in higher education right before the outbreak of the pandemic, with HEIs developing customized medium- and long-term strategy plans focusing on sustainability. However, the COVID-19 pandemic has halted several initiatives and strategic plans. Most HEIs were compelled to adjust their focus from 'sustainability' to 'survival' as a result of the pandemic influence, blocking the path of transformative change. If sustainability is to be revived as a development objective, then during pandemics, the prerequisite condition for HEIs is the development of effective plans and policies that attract new enrolments, fill seats to capacity (full resources), and raise revenues, without which sustainable system may be hindered to obtain desire effects (302). As sustainability relates to the capability of being supported or maintained or kept going (303), it must be reviewed during pandemic.”

***Please refer line numbers 620-631 in the revised manuscript highlighted in RED TEXT.***

**Comment (2)**

In the methodology describe in brief statistical methods used in the paper.

**Response by authors**

As per reviewer’s suggestion following text is added

“To validate hypothesis H1, Analysis of Variance (ANOVA) was utilized to identify the significant difference in students’ perceptions of sustainability of ECs under the COVID-19 pandemic. Hypothesis H2 was tested by Regression Analysis to detect the accountable relationship in between the sustainability of ECs under COVID-19 pandemic and the ECs’ characteristics. ANOVA was performed to predict the statistical significant across the students’ characteristics to explain regression model representing the relationship in between the sustainability of ECs under COVID-19 pandemic and the ECs’ characteristics to justify Hypothesis H3. All above statistical analysis were performed by Statistical Product and Service Solutions (SPSS).”

***Please refer line numbers 787-795 in the revised manuscript highlighted in RED TEXT.***

Once again thank you!

AUTHORS
